# How the brain controls urination

**DOI:** 10.7554/eLife.33219

**Published:** 2017-12-04

**Authors:** Anna P Malykhina

**Affiliations:** Division of Urology, Department of SurgeryUniversity of Colorado Anschutz Medical CampusAuroraUnited States

**Keywords:** bladder, micturition, locus coeruleus, Barrington's nucleus, cortex, voiding, Rat

## Abstract

Coordination between the brainstem and the cortex helps to ensure that urination occurs at an appropriate time.

**Related research article** Manohar A, Curtis AL, Zderic SA, Valentino RJ. 2017. Brainstem network dynamics underlying the encoding of bladder information. *eLife* **6**:e29917. doi: 10.7554/eLife.29917

The brain and the bladder must communicate to make sure that we only urinate when and where it is appropriate. The process of urination is partly controlled by reflexes and is partly under conscious control (de Groat et al., 2015). As the bladder fills, it sends sensory information to the central nervous system, and when the bladder is full, these signals indicate that it must be emptied soon.

One of the signals going the other way, from the brain to the bladder, is the activation of a part of the brainstem called the PMC, short for pontine micturition center. (The word 'micturition' originally referred to the urge to urinate, but is now often used to describe the process of urination as well). The PMC connects to other centers in the central and peripheral nervous systems to coordinate when urination occurs (Fowler et al., 2008). Many studies have identified and examined the main brain centers involved in the control of urination. However, the connections between these different centers, including when and for how long they become active, remain elusive. It is also unclear how the cortex – the part of the brain responsible for higher thought processes – influences urination.

Now, in eLife, Rita Valentino of the Children’s Hospital of Philadelphia and co-workers – including Anitha Manohar as first author – report how neuronal activity is orchestrated before, during and after urination in rats (Manohar et al., 2017). The researchers evaluated when and where neurons fired in unanesthetized rats as their bladders filled and then emptied by recording neural activity in three regions of the brain that are involved in urination: the PMC, the locus coeruleus, and the medial prefrontal cortex (mPFC; Figure 1). At the same time, they measured both the pressure within the bladder and the frequency of urine output.

**Figure 1.**
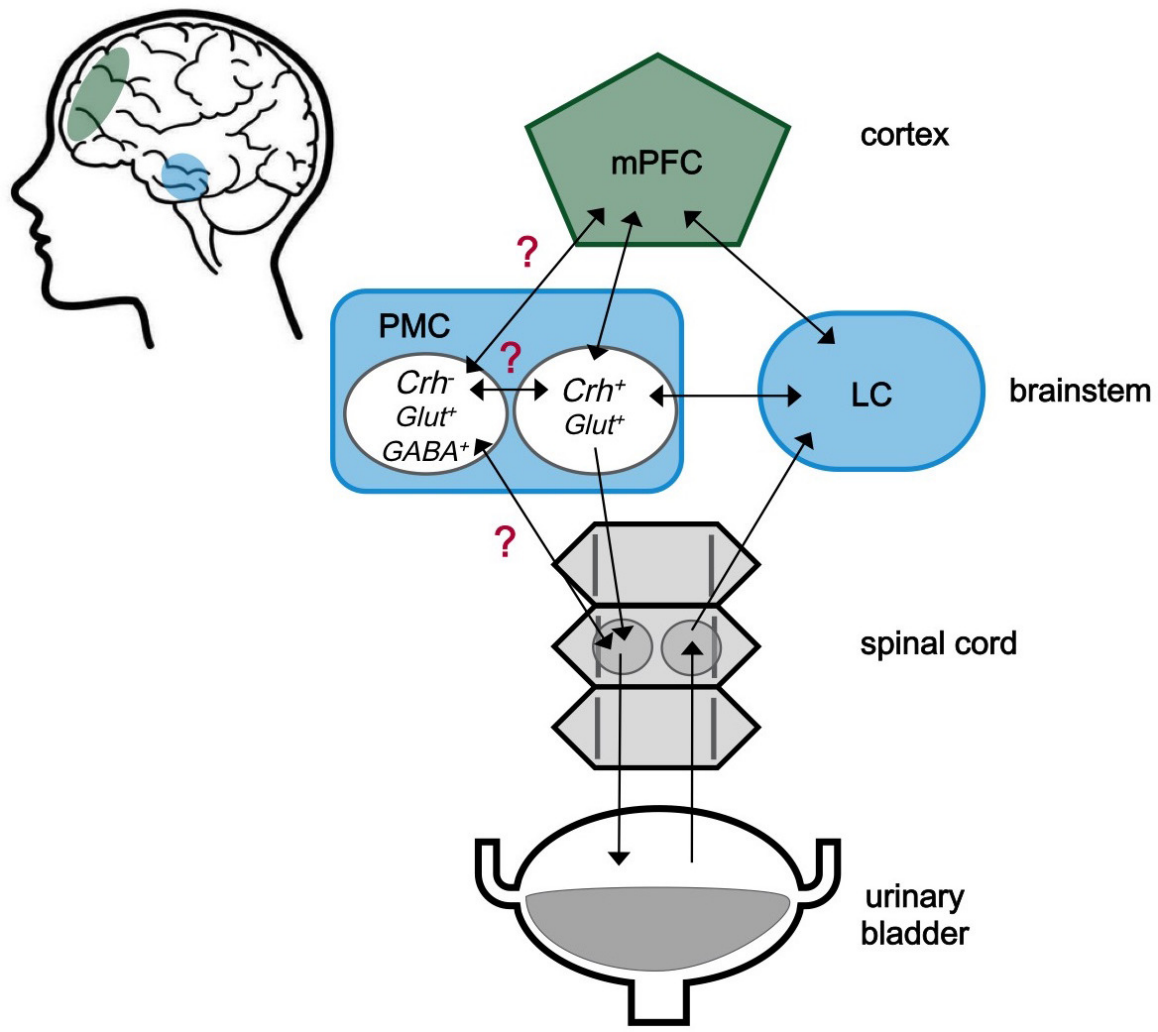
The neuroscience of urination. The medial prefrontal cortex (mPFC) is located behind the forehead at the front of the cortex (green), while the pontine micturition center (PMC) and the locus coeruleus (LC) are located within a part of the brainstem known as the pons (blue). The approximate locations of these regions within the human brain are shaded in the cartoon on the left. These three brain regions both send and receive signals (represented by arrows) to and from each other. Signals from the bladder are relayed via the spinal cord to the LC, and then to other centers in the brain including the PMC. The PMC sends signals to the bladder via the spinal cord. The PMC contains different kinds of neurons. Neurons expressing corticotropin-releasing hormone are labeled Crh^+^, and are known to be involved in starting urination. Neurons that do not express this hormone are labelled Crh^-^. The role of these neurons is less clear, but it is possible that they are involved in urine storage. Glut^+^ and GABA^+^ indicate neurons that produce glutamate and GABA, respectively. Confirmed connections with unclear effects are marked with a red question mark.

First, Manohar et al. established that all neurons in the PMC show the same firing patterns, characterized by slow background activity and fast bursts during the intervals between urinations. These bursts became rare prior to urination, more prominent during urination, and continued for several seconds after the bladder had been emptied. This timing of neuronal activity suggests that PMC neurons likely play a more complex role in regulating the emptying of the bladder than a simple 'on-off switch'. The bursts of activity in the PMC during the intervals between urinations were also intriguing. It was previously believed that PMC neurons were only active during urination (Betts et al., 1992).

In the seconds before urination, neurons in the locus coeruleus showed ongoing low frequency bursts with stronger theta oscillations (waves of activity that repeat about seven times per second). At the same time, the activity in the locus coeruleus started to more closely match that in the mPFC, though the activity across the mPFC became less synchronized. It is likely that some of these changes help to begin the process of urination by increasing arousal and shifting attention toward the full bladder (Michels et al., 2015).

The relationship between the locus coeruleus (LC) and the PMC is also interesting. These centers are close enough that neuronal activity could be recorded from both regions at the same time. The results showed that LC neurons were activated before PMC neurons, suggesting that the former receives indirect input from the bladder before the stimulus reaches the PMC. This is consistent with earlier observations using fMRI in rats (Tai et al., 2009).

The new findings reported by Manohar et al. raise a few questions. The roles of the brainstem and the cortex in processing information from the bladder, and in coordinating urination, remain unclear. It is also not obvious why PMC neurons show bursts of activity in the intervals between urinations. Recent data suggest that there are different kinds of neurons in the PMC (Figure 1), so it is possible that a specific population of PMC neurons sends signals that help the bladder store urine by releasing different types of neurotransmitters (Hou et al., 2016). Earlier studies also showed that urine storage reflexes are mainly organized in the spinal cord (Drake et al., 2010). However, a group of neurons located in the brainstem might play a role in urine storage too. When activated, these neurons made the external urethral sphincter – the muscle that allows us to choose to start urination – more active (Blok and Holstege, 1999).

We need more data about the pathways through which the locus coeruleus receives information from the bladder before it is transmitted to the PMC. Future experiments should also explore what other regions of the cortex get synchronized or desynchronized during urination. Manohar et al. speculate in these areas, based on the published literature, but further studies are clearly warranted to provide more definitive answers.
